# Policy and stakeholder analysis of infant and young child feeding programmes in Sri Lanka

**DOI:** 10.1186/s12889-017-4342-4

**Published:** 2017-06-13

**Authors:** Sanjeeva S. P. Godakandage, Upul Senarath, Hiranya S. Jayawickrama, Indika Siriwardena, S. W. A. D. A. Wickramasinghe, Prasantha Arumapperuma, Sathyajith Ihalagama, Srisothinathan Nimalan, Ramanathan Archchuna, Claudio Umesh, Shahadat Uddin, Anne Marie Thow

**Affiliations:** 1 Family Health Bureau, Ministry of Health, Nutrition and Indigenous Medicine, Colombo, Sri Lanka; 2 Faculty of Medicine, University of Colombo, Colombo, Sri Lanka; 3 Ministry of Technology and Research, Colombo, Sri Lanka; 4 Ministry of Health, Nutrition and Indigenous Medicine, Colombo, Sri Lanka; 5 Epidemiological Unit, Ministry of Health, Nutrition and Indigenous Medicine, Colombo, Sri Lanka; 6 South Asia Infant Feeding Research Network, Colombo, Sri Lanka; 7 Complex Systems Research Group, University of Sydney, Sydney, Australia; 8 Menzies Centre for Health Policy, School of Public Health, University of Sydney, Sydney, Australia

**Keywords:** IYCF, Policy, Stakeholder analysis, Net-map

## Abstract

**Background:**

Infant and young child feeding practices (IYCF) play a critical role in growth and development of children. A favourable environment supported by appropriate policies and positive contributions from all stakeholders are prerequisites for achieving optimal IYCF practices. This study aimed to assess the IYCF-related policy environment and role of stakeholders in policy making in Sri Lanka, in order to identify opportunities to strengthen the policy environment to better support appropriate IYCF and reduce childhood malnutrition.

**Methods:**

We mapped national level policy-related documents on IYCF, and conducted a stakeholder analysis of IYCF policy making. A matrix was designed to capture data from IYCF policy-related documents using a thematic approach. A narrative synthesis of data from different documents was conducted to achieve the first objective. We then conducted an analysis of technical and funding links of stakeholders who shape IYCF policies and programmes in Sri Lanka using the Net-Map technique, to achieve the second objective. A total of 35 respondents were purposively selected based on their knowledge on the topic, and individual interviews were conducted.

**Results:**

Twenty four policies were identified that contained provisions in line with global recommendations for best-practice IYCF, marketing of breast milk substitutes, strengthening health and non-health systems, maternity benefits, inter-sectoral collaboration, capacity building, health education and supplementation. However, there is no separate, written policy on IYCF in Sri Lanka.

Participants identified 56 actors involved in shaping IYCF policies and programmes through technical support, and 36 through funding support. The Government Health Sector was the most connected as well as influential, followed by development partners. Almost all actors in the networks were supportive for IYCF policies and programmes.

**Conclusions and recommendations:**

All evidence-based recommendations are covered in related policies. However, advocacy should be targeted towards strategic support for IYCF in high-level policy documents.

The stakeholder analysis confirmed a network led by the government health sector. Enhancing the multi-sectoral commitments stressed in policy documents is an opportunity to strengthen IYCF policy process in Sri Lanka.

**Electronic supplementary material:**

The online version of this article (doi:10.1186/s12889-017-4342-4) contains supplementary material, which is available to authorized users.

## Background

Even though improvement of child nutritional status has been identified as a priority by the Ministry of Health, available data suggests that indicators related to the child nutrition in Sri Lanka have not improved on par with other health indicators. According to Demographic and Health Survey (DHS) 2006/7, the prevalence of underweight among children under five was 22%, a marginal decrease from 23% in year 2000 [1]. Stunting in the same age group was 17.3% in 2006/7, again similar to the prevalence of 18% in year 2000 [1].

This is in the backdrop of a well-established service delivery structure for Infant and Young Child Feeding (IYCF) care. The health sector in Sri Lanka comprises curative and preventive services. In the curative sector, there are specialist and non-specialist government hospitals, in addition to few private hospitals concentrated in urban areas. In the preventive health sector, the frontline healthcare worker delivering maternal and child health services is the Public Health Midwife. There is a Public Health Midwife for an average population of 3000, who is responsible for IYCF care. Services are delivered by her during home visits, as well as within clinic settings. Public Health Midwife receives technical support from her immediate supervisor, the Medical Officer of Health, who is a graduate medical officer appointed to an area of approximately 60,000 population. Healthcare delivery is devolved to the nine Provinces, yet policy level decisions are taken at National level.

Effective interventions for improving IYCF practices are well established – key components are: promotion of breastfeeding (including counselling); strategies to promote complementary feeding (including counselling, food supplementation and food based comprehensive approaches); micronutrient interventions; and general supportive strategies to improve family and community nutrition [2–4]. For the best outcomes at a national level, such interventions need to be supported by appropriate policies, including policies from sectors other than health [5]. However, many low and middle-income countries (LMIC) have limited policy support for IYCF. In 2013, Gupta and colleagues found that, of the 40 countries assessed, Sri Lanka had a good policy and programme support in essential areas for the improvement of IYCF. However, gaps were identified in relation to maternity protection and information support [6, 7].

This study was designed to assess the IYCF-related policy environment, and the role of stakeholders in policy making in Sri Lanka, in order to identify opportunities to strengthen the policy environment to better support appropriate IYCF, and to reduce childhood malnutrition. The current study is a part of the research project on IYCF policies by South Asian Infant Feeding Research Network (SAIFRN) researchers in Bangladesh, India, Nepal, Pakistan and Sri Lanka.

## Methods

### Policy mapping

The first component of this study mapped national level policies and policy-related documents providing support for IYCF in Sri Lanka across four domains of best-practice interventions, and focussed on caregivers/mothers:

1. Policies and guidelines etc. that provides general policy support for infant and young child feeding
2. Policies and guidelines etc. that supports provision of evidence based information to mothers/caregivers and general public on IYCF
3. Policies and guidelines etc. that supports training of healthcare workers to enhance their capacity to provide IYCF services
4. Policies and guidelines etc. that enables mothers/caregivers to engage with best practice interventions

The SAIFRN study team used mind mapping/causal analysis to identify 1) relevant policy sectors that could incorporate or support IYCF, and 2) relevant types of policy interventions, ranging from ‘high level’ policies that provide strategic guidance for policy making (indicating political interest/will), to sector-specific policies (such as health sector policies) and more focussed implementation-relevant strategies and guidelines. A brainstorming session was conducted by the team members and prepared a causal analysis diagram to identify relevant policy sectors, and based on it a mind map diagram was drawn to identify relevant policy documents. The mind mapping process allowed ideas to be generated in a loosely structured brainstorming session. As the ideas were generated, the policy documents were informally categorized by their placement in one of the policy category branches. At the end of the brainstorming session, the mind map was reviewed for completeness.

Documents were identified by searching government websites and government archives, and through requests to Ministry of Health officials, and those from related Ministries. In order to achieve comprehensiveness, we crosschecked the list of documents with national level experts in IYCF as well as nutrition.

With respect to each policy or policy-related document, we recorded details of policy support for IYCF in a predetermined matrix in Microsoft Excel, including key document details, policy type and text relevant to each of the domains listed above. We analysed the data using a narrative synthesis approach, focussing on:

- Presence of IYCF in policy documents
- How comprehensive the approach is (e.g. different sectors, avenues)
- Translation of high-level policy statements into implementation-relevant documents

Both the content analysis of the matrix and thematic analysis were conducted initially by a Research Assistant, which were independently crosschecked by an investigator.

### Stakeholder analysis of IYCF policies and programmes

The Stakeholder analysis was carried out using Net-Map, a low cost interview-based mapping tool which allows to determine linkages, influence and goals (measured as support) of the stakeholders involved in a situation [8].

### Identification of the study population and recruitment of respondents

A preliminary list of potential interviewees – those known to be knowledgeable about IYCF policy and programmes in Sri Lanka – was identified by the research group from a range of relevant organisations. Each team member ranked the potential respondents of the preliminary list independently. The interviews were initiated with the highest ranked respondent and continued with others. Participants were sent written invitations through their institutional heads where applicable. Additional respondents were identified through snowball sampling, with each respondent asked to identify other potentially relevant respondents.

### Development of the study guide

The SAIFRN policy study group developed a detailed interview guide, based on Net-Map methodology, with assistance from Net-Map trainers from the International Food Policy Research Institute. Pre-testing of the tool was carried out with a Medical Officer at Family Health Bureau with a long-term experience in IYCF related activities. Prior to initiation of data collection, each interviewer conducted at least two pre-tests – one with a member of the research team and the other with a trusted outsider.

### Conduct of interviews

Before the initiation of the interview, respondents were informed about the techniques and objectives of the study. Verbal consent was obtained. Interviews were conducted on an individual basis by trained interviewers, and asked about: actors that influence policies and programs for IYCF, the links between actors (both technical and funding), level of influence, and level of support for IYCF.

Detailed qualitative data was also collected from the discussion amongst participants. When participants identified a potential actor, link or level of influence, interviewers asked probing questions to identify the reasons behind each given decision. Interviews were conducted until no new information was generated from each new interview.

The research was approved by the Ethics Review Committee of the Faculty of Medicine, University of Colombo (Ref. No: EC-13-031), and by the Secretary, Ministry of Health, Sri Lanka.

### Analysis of net-map data

Interview data regarding actors, technical and funding links, level of influence and level of support were entered into ORA (Organizational Risk Analyzer; copyright Carley, Carnegie Mellon University) [9]. This software was then used to generate graphical representation of individuals (or organizations) and their relationships.

The relative importance of an actor in social network is determined by several centrality measures including in-degree, out-degree, closeness and betweenness. In-degree is a measure of receptivity of an actor node, and relates to the number of ties directed towards the node. Out-degree is the number of ties the node directs towards other nodes, and is a measure of expansiveness or activity of an actor in the network. Betweenness is the sum of times an actor node appears along the shortest path between two other actors. This centrality measure represents the capacity of an actor to control the flow of information between any pair of all other member actors in a network [10]. Closeness measures the shortest paths between a node and all other nodes, and represents the reachability of an actor from the other actors in a network [10].

Other than the above centrality measures, the level of influence and support of each actor were measured as independent attributes. Influence was defined as the ability of an actor to influence, and not merely the formal hierarchies. Support is whether an actor supports or opposes the process or the direction they want to move [8]. We used the qualitative data collected during the Net-Map interviews to help to explain the quantitative data.

Using the community detection algorithm of ORA software, communities were identified in both technical and funding networks. A community indicates that nodes belonging to that community have more connections or collaborations among themselves (in terms of technical support or funding), compared to their connections with the nodes belong to other communities.

## Results and discussion

### Content analysis

We mapped national level policies and policy-related documents that provide support for IYCF in Sri Lanka. A total of 24 policies and policy-related documents on IYCF were analysed, 20 of which were from the health sector. The Ministry of Public Administration and Home Affairs and the Ministry of Labour were the other ministries that had policy documents related to IYCF.

Overall, policy support for IYCF was evident across all four domains studied (1.Policies and guidelines etc. that provide general policy support for infant and young child feeding; 2. Policies and guidelines etc. that support provision of evidence based information to mothers/caregivers and general public on IYCF; 3. Policies and guidelines etc. that support training of healthcare workers to enhance their capacity to provide IYCF services; 4. Policies and guidelines etc. that enable mothers/caregivers to engage with best practice interventions). Some of the miscellaneous provisions that do not fit into above four domains were categorised as ‘other’. More details on policy documents across four domains are described below.

### General policy support for infant and young child feeding

Under the general policy support, we reviewed higher-level policy documents that provide policy support for health sector as well as other relevant sectors. Ten policy documents were found to have provisions aimed at prioritising IYCF, and strengthening healthcare and related systems to deliver IYCF related services [11–20]. However, there was no mention of IYCF in ‘high level’ policies that indicate political will and provide strategic guidance for policy making [20].

At a sectoral level, the Family Health Bureau within the Ministry of Health is the national level body responsible for IYCF services, whilst the Nutrition Coordination Division liaises with all the relevant sectors. The National Strategic Plan on Maternal and New-born Health, and the Multi Sector Action Plan on Nutrition contain provisions on strengthening these two institutions respectively [12, 13].

One of the key areas for general IYCF policy support, as mentioned in National Nutrition Policy and National Strategic Plan for Maternal and New born Health, was to “strengthen partnerships and networking with relevant sectors and stakeholders … to improve nutrition at community level”, and to improve collaboration for maternal and child health [11, 13], in line with the Innocenti Declaration [21]. There are also specific recommendations within the health sector, such as to institute multisectoral mechanisms to improve coherence in IYCF-related policy making, including establishing a Nutrition Coordination Division within the Nutrition Secretariat by the Multi-sector Action Plan for Nutrition [12], and to “establish a mechanism for regular consultation and dialogue between political leadership, policy planners and other stakeholders to ensure sustainability of programmes” by the National Nutrition Policy [11].

General policy support identified by above policies was pursued by more specific policies, majority of which are health sector specific as described below.

### Provision of evidence based information to mothers/caregivers and general public on IYCF

Policies to support the provision of correct information to mothers/caregivers exist in the form of clear national recommendations regarding appropriate IYCF that guides general public awareness campaigns, legal provisions to prevent distribution of information by manufacturers of breast milk substitutes, and provision of counselling to caregivers (Additional file 1).

There are clear and consistent, evidence based policy provisions regarding appropriate IYCF in the National Maternal and Newborn Health Policy, and the Sri Lanka Code for the Promotion Protection and Support of Breast Feeding and Marketing of Designated Products (Amended 2002) [14, 15]. These policies form a strong basis for messages communicated to mothers/caretakers and the general public.

The above provisions are clearly transferred to implementation level documents. The Infant and Young Child Feeding Guidelines facilitate the implementation of these policy recommendations by specifying the messages that need to be communicated [22]. Within the health sector, information for mothers on current evidence based recommendations on breastfeeding are included in the circular ‘Breastfeeding: Just 10 Steps: The Baby Friendly Way’ [18]. The Sri Lanka Code contains the previous recommendation on breastfeeding initiation within thirty minutes of birth, but the updated circular ‘Breastfeeding: Just 10 Steps: The Baby Friendly Way’ conforms to the new recommendation on helping mothers to initiate breastfeeding within one hour of birth [15, 18].

There is strong policy support in the Multisector Action Plan on Nutrition, for general public awareness. This includes a national advocacy and communication campaign for improved maternal and child nutrition with a focus on the first 1000 days [12], which includes the specific implementation guidance: “effective national mass media communication campaign to promote optimal maternal nutrition & Infant and Young Child Feeding”. This partially fulfils the Global Strategy for Infant and Young Child Feeding, which stresses ensuring that all who are responsible for communicating with the general public, including educational and media authorities, provide accurate and complete information about appropriate IYCF practices.

Ten of the policies and policy-related documents [11–15, 17–19, 22, 23] contained provisions related to IYCF counselling, in line with evidence that ‘one to one counselling’ and ‘group counselling’ have the potential to increase age appropriate exclusive breastfeeding significantly, and that complementary feeding can be enhanced through education programmes and counselling [3, 24, 25].

With regard to legal provisions, the Sri Lanka Code emphasises the responsibility of healthcare and other community workers to inform the public regarding the advantages of breastfeeding, without any interference from manufacturers of breast milk substitutes or complementary foods [15]. Healthcare workers are also protected by the Code, which also states that no information regarding IYCF shall be given by any manufacturer or distributor to a healthcare worker [15], in line with the International Code of Marketing of Breast milk Substitute [26].

At a broader level, the National Strategic Plan on Maternal and Newborn Health emphasises strengthening accessibility, availability and utilization of Maternal Child Health services, and ensuring a functioning quality assurance system in clinic settings [13]. The Protocol on Managing Nutritional Problems among Under- five Children in the Community, and the circular on ‘Change in the modality of Management of Children under the age of 5 years with Severe Acute Malnutrition’ empower the grassroots level healthcare workers to provide correct information to mothers/caregivers by clearly outlining the referral conditions, and pathways for deviations from normalcy identified in the growth monitoring and promotion process [16, 27].

In reviewing the policy documents using the matrix, it was evident that there was a lack of policy support in the education sector regarding the provision of correct information on IYCF. This suggests an opportunity to strengthen proactive policy support that would reach the next generation of mothers in Sri Lanka through school based education.

### Training of healthcare workers to enhance their capacity to provide IYCF services

We observed that capacity building took three forms: in-service training, pre-service education, and professional development courses, in line with global recommendations [28]. The National Nutrition Policy provides recommendations for “building capacity of health staff and community–based workers for effective behaviour change communication with regard to nutrition promotion”, and the National Strategic Plan on Maternal and Newborn Health mentions overall human resource development on maternal and child health [11, 13] (Additional file 1). Similarly, at the implementation level, the circular “Breastfeeding: Just 10 Steps: The Baby Friendly Way” stresses that all local health workers should have appropriate breastfeeding support training, and the Multi Sector Action Plan for Nutrition more specifically mentions capacity building of relevant health staff on IYCF counselling and communication [12, 18]. These policies are in line with global recommendations for pre-service and in-service training.

An adaptation of UNICEF/WHO Lactation Management Training manual has been at the centre of Sri Lanka’s efforts to promote and support breastfeeding [29], and since 1995, a large number of healthcare workers have undergone training using that manual.

### Enabling mothers/caregivers to engage with best practice interventions

We identified 16 policy documents that provide support for appropriate IYCF by enabling mothers and caregivers to engage in age-appropriate feeding, and with best practice interventions [11–15, 17–20, 22, 23, 30–34] (Additional file 1).

The availability of maternal and child health services are prioritised under the National Strategic Plan on Maternal and New-born Health, which provides for ‘mechanisms for mobilization and utilization of funds for maternal and child health/family planning from government and other sources at central, provincial and district levels’ [13].

Mothers are also enabled to engage with healthcare services through provisions in the Code to strengthen the healthcare system to improve IYCF services, such as ‘to enable mothers to provide optimal care including exclusive breastfeeding for 6 months and continuation of breastfeeding for 2 years and beyond’, and the ‘healthcare system shall encourage, protect and support breastfeeding and cooperate with government authorities in giving effect to the provisions of the code’ [15]. The National Maternal and Child Health Policy is more specific in this regard, and recommends implementing evidence-based interventions; specifically ensuring exclusive breastfeeding for 6 months, followed by appropriate complementary feeding, together with continuation of breastfeeding for 2 years and beyond, and regular growth monitoring and promotion. Support for good IYCF practices is further reinforced by the requirement in National Maternal and Child Health Policy that ‘every hospital providing maternity services and care for newborn infants having a written breastfeeding policy’ [14], in line with the highly successful Baby Friendly Hospitals Initiative [28].

Mothers are also supported at the community level through referral to breastfeeding support groups in line with Step 10 of the ‘Baby-friendly Hospital Initiative’ (i.e. to foster the establishment of breastfeeding support groups and refer mothers to them on discharge from hospital or clinic). Mothers are also protected from promotion of breast milk substitutes and complementary foods for infants less than 6 months, with the provision of free samples or supplies of designated products or complementary foods to mothers or their family members or to the general public not being permitted under the Sri Lanka Code [15].

In Sri Lanka, service delivery is also strengthened through specific policy support for monitoring and evaluation of nutrition programmes through the National Nutrition Policy [11] and the National strategic plan on Maternal and Newborn Health (2011), in line with the Innocenti declaration that provides recommendations regarding monitoring and evaluation of programmes [13, 21].

Unsupportive work environments, such as limited or no maternity leave, inflexible working hours and lack of breastfeeding breaks, unavailability of breastfeeding rooms or space for expressing and storing breast milk, and limited access to services are contributory factors for IYCF and nutritional problems [28]. Maternity leave is recommended under the Sri Lanka Code for Protection, Promotion and Support of Breastfeeding and Marketing of Designated Products, and also supported in the Guidelines on Infant and Young Child Feeding [15, 22]. However, disparities were observed among policy documents that address different sectors. Specific policies that provide for maternity leave are the Shop and Office Employees Act, which recommends leave for 28 days, and the circular ‘Maternity Leave - Chapter XII of the Establishments Code’ for public officers that ensures 84 working days of leave for every live childbirth [30, 32]. Further, these provisions do not cover mothers who are not public officers, and employees engaged in ‘atypical forms of dependent work’ are not covered in Sri Lankan maternity benefits recommendations. Paternity leave of three working days is provided for in the circular ‘Paternal Leave - Chapter XII of the Establishments Code’ [33]. These gaps have been highlighted by the World Breastfeeding Trends initiative assessment report as well. Further, the Multi-sector Action Plan on Nutrition calls for a review of the existing regulations on maternity leave [12].

In addition to the recommendations for maternity leave, the circular “Facilitation of Practice of National Infant and Young Child Feeding (IYCF) recommends the establishment of Breastfeeding Rooms within health institutions [31].

### Other

We also identified policy support for IYCF in the form of actions to provide supplementation when necessary, support food safety, provide appropriate complementary foods, ensure equity in relation to IYCF and provisions to ensure good IYCF practices in emergencies, all of which are in line with global recommendations [3, 5, 6, 35] (Additional file 1).

Food security is supported in the National Nutrition Policy (2008): “access to adequate, nutritious, safe and quality food at affordable price throughout the year” [11]. Food safety for complementary foods is mandated under the Sri Lanka Code, which states that manufacturers of any designated product or complementary food, or any person acting on his behalf shall conform to the quality control standards and procedures, and the codes of hygienic practices for foods and other related products for infants and young children, in line with the International Code of Marketing of Breast-milk Substitutes [15, 26].

Policy support for micronutrient supplementation for young children includes the Multiple Micronutrient Supplementation Programme (to be scaled up under the NNP and Multi-sector Action Plan for Nutrition), the implementation of which is addressed in the ‘Guidelines on Infant and Young Child Feeding’, the ‘Protocol on Managing Nutritional Problems among Under Five Children in the Community’ and the circulars “New Schedule for Multiple Micro Nutrient (MMN) supplementation for 6, 12 and 18 Month Age Groups”, and “Vitamin A mega dose supplementation – revised schedule” [11, 12, 22, 27, 36, 37]. The Guidelines on Infant and Young Child Feeding of Sri Lanka recommends using fortified complementary foods (Thriposha) to infants with growth faltering or underweight [22]. Specific information on implementation is provided in the ‘Protocol on Managing Nutritional Problems among Under Five Children in the Community’ [27].

We also identified a focus on equity in the relevant policy documents reviewed in Sri Lanka. The Multi Sector Action Plan for Nutrition has provisions for “nutritionally vulnerable children under 2 years”, and “Crèches and pre-schools in plantations below 250 Acres” specially been addressed [12]. The National Nutrition Policy recommends targeting “underserved areas, plantation community, urban poor and areas identified by the nutrition surveillance system” [11], whilst the National Strategic Plan on Maternal and Newborn Health highlights “post-conflict, vulnerable, underserved, under-privileged, marginalized and internally displaced populations” [13].

Finally, we also identified provisions on infant feeding in emergencies. The December 2004 tsunami found Sri Lanka without sufficient guidelines on infant feeding in emergencies, and the national guidelines on infant feeding during emergencies were formulated [17]. The Sri Lanka Code states that during emergencies bottles and teats should never be distributed and their use should be discouraged, and also stipulates appropriate use of Breast Milk Substitutes [15]. This is in line with international recommendations for supporting appropriate infant and young child feeding in emergencies [15, 26].

### Stakeholder network analysis

Altogether 35 interviews were conducted with officers knowledgeable about IYCF policy and programme process in Sri Lanka, with a response rate of 100%. The majority of the respondents were from the government health sector (Table 1).

**Table 1:** Basic statistics of the interviewees

Category	Number interviewed
Government Sector- Health	20
Government sector –Non Health	02
Research and Academic	05
Development partner	05
NGO /Civil Society	02
Other	01
Total	35

**Fig. 1:**
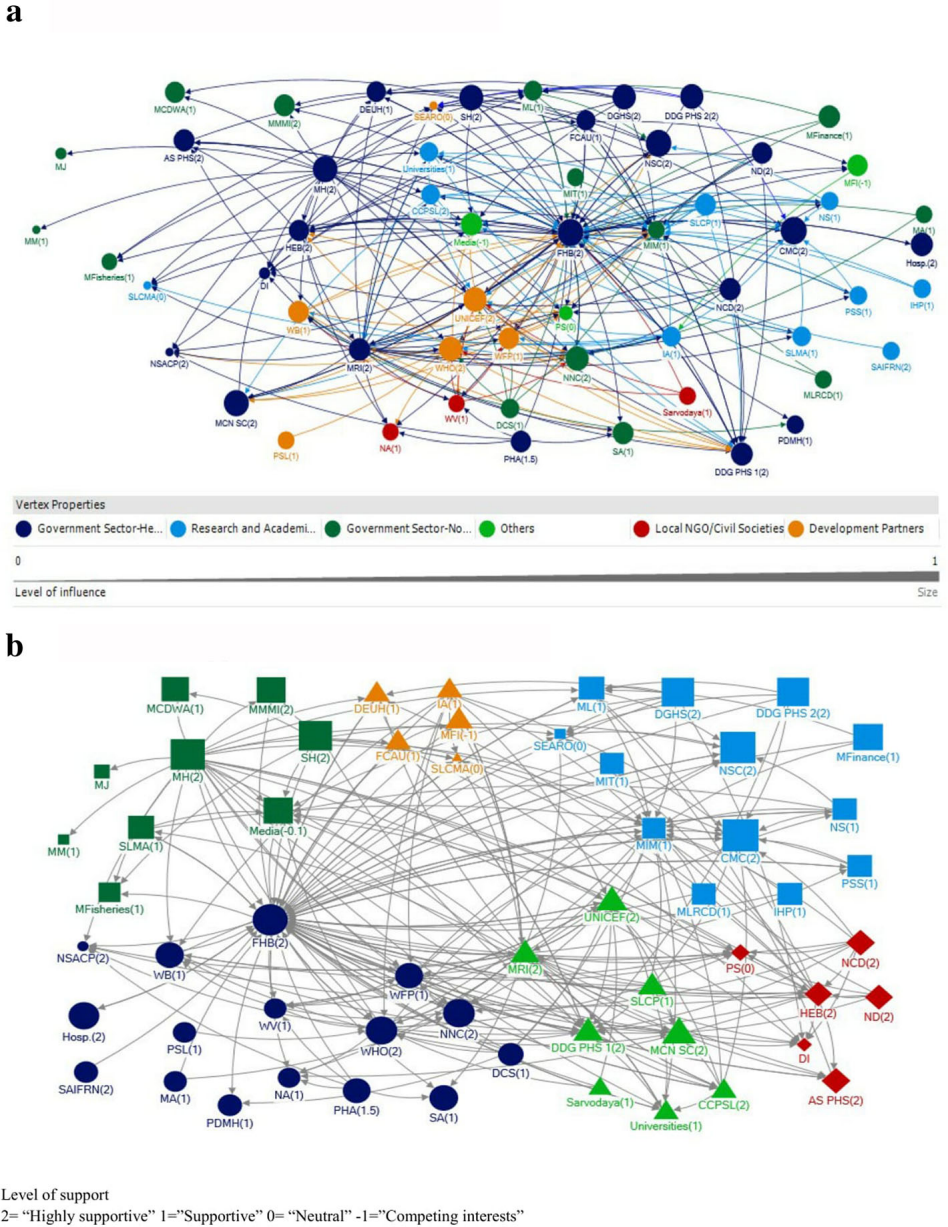
Technical Support Network of the IYCF polocy and programme process in Sri Lnaka; by sectors (**a**) and communities (**b**)

**Fig. 2:**
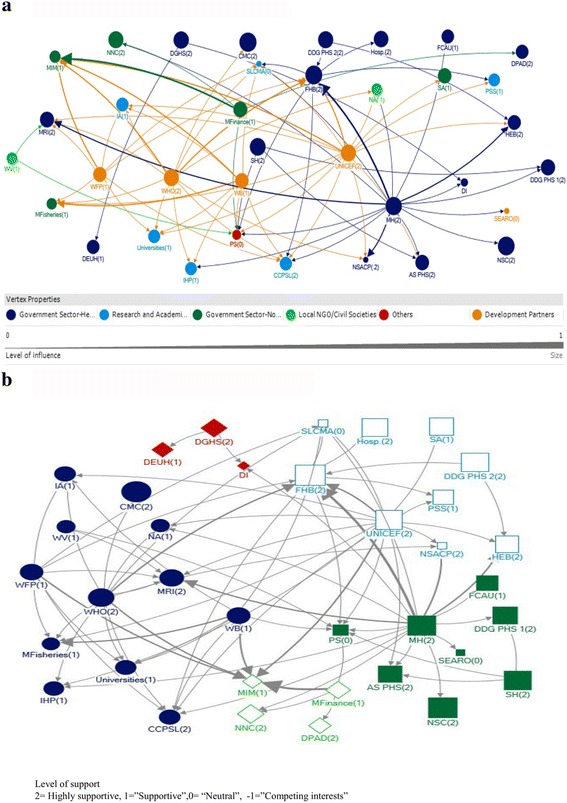
Funding support network of IYCF  policy and programme process in Sri Lanka; by sectors (**a**) and communties (**b**)

The network analysis of technical and funding support in Sri Lanka, based on the above interviews, revealed influential actors from five main sectors namely, government health, government non-health, development partners, research and academic sector and local NGO sector, as influential in IYCF policy decision making. An actor is indicated by a node in the network, and actors from the same sector are given the same colour. The directions of the linkages between actors (nodes) are marked as arrows. Node size is proportionate to the level of influence (ability of an actor to influence other actors), and numbers in brackets indicate the median level of support each actor exerts towards the shaping of IYCF policies and programmes in Sri Lanka (Figs. 1 & 2).

### Stakeholders in the technical support network their position in the network, level of influence and support

All the actors who provide technical support for the IYCF policy and programme process in Sri Lanka and their linkages are displayed in the technical support network. The technical support network for IYCF policy and programmes in Sri Lanka consists of 57 identified actors and the connections among them (Fig. 1a; Centrality measures in Additional file 2). Most of the actors in the technical support network were from the government health sector.

When analysing the technical linkages of government health sector actors, most of them were among the 20 actors that received substantial technical assistance. They received technical inputs from a wide range of other organisations including development partners, research and academic sector (Fig.1-a; in-degree centrality in Additional file 2) which enabled them to take well-informed technical decisions. Further, most of the actors from government health sector were among the top 20 actors for providing technical support to other organizations in the network (Fig.1-a; Out-degree centrality in Additional file 2). Similarly most of the government health sector actors were among the 20 actors who had the highest capacity to control the flow of the information (Betweenness in Additional file 2, Fig. 1a) and highest reachability (Closeness in Additional file 2, Fig. 1a). Government health sector was the most influential sector in the policy and programme process in the Sri Lanka. Out of all the actors from the government health sector, more than 80% were ‘highly supportive’ and the rest were ‘supportive’ (Additional file 3). The first eight actors with the highest level of influence were from the government health sector (Additional file 4).

The Family Health Bureau (FHB) was the most influential actor with respect to technical support (Additional file 4), and was also highly supportive (Additional file 3). It ranked highest for receiving technical support (in-degree), providing technical support (out-degree), being accessible to other actors in the network (closeness), and being influential over the flow of information between actors by its position in the network (betweenness) (Fig. 1a, Additional file 2). Another supportive and influential government health agency was the Health Education Bureau (HEB), which ranked in the top ten for provision of technical support (out-degree), and its strategic position in the network (betweenness and closeness) (Additional file 2, Additional file 4, Additional file 3). The Code Monitoring Committee was identified as the actor with the highest level of influence in the technical support network, followed by the FHB, which the participants noted due to its legal authority (Additional file 4).

The National Nutrition Council (NNC), which is a Ministerial level council headed by His Excellency the President, was also seen as highly influential and very supportive, and ranked in the top ten actors for receiving technical support (mainly from local NGOs and development partners) (Additional file 2, Fig. 1a). Participants identified the NNC as an important body for nutrition and IYCF since representatives from all relevant ministries, not only the Ministry of Health, are members – thus providing high-level recognition that nutrition is a multi-sectoral issue. Provincial health authorities were among the top 20 actors for out degree. Further, they had adequate amount of betweenness and closeness, which enable the flow of technical expertise from central level to provincial level, as well as from provincial level to central level.

When analysing the technical linkages of development partners, it was revealed that most of them also received technical inputs from a large number of other actors from different sectors (In-degree in Additional file 2, Fig. 1a), which facilitated their functioning in the Sri Lankan context. Most of them were also among the top 20 actors for providing technical inputs (Out-degree in Additional file 2, Fig. 1a), which facilitated the flow of international expertise to the IYCF programme and policy process in Sri Lanka. Most of the development partners were among the 20 actors who had the highest capacity to control the flow of the information (Betweenness in Additional file 2, Fig. 1a) and highest reachability (Closeness in Additional file 2, Fig. 1a). Further, they were found to be influential actors (Fig. 1a; Additional file 4) and most of them were ‘supportive’ or ‘highly supportive’ to the IYCF programme and policy process in Sri Lanka (Additional file 3). The strategic position of development partners in the network and high level of influence combined with high level of supportiveness was seen as ensuring the flow of international expertise into the IYCF policy and programme process in Sri Lanka.

The Net-Map analysis of research and academic sector revealed two groups of researchers/academics who were influential actors in provision of technical support (out-degree, Table 1) for IYCF in Sri Lanka. The College of Community Physicians in Sri Lanka (CCPSL) and several Independent Academics (IA) were identified by participants, and qualitative discussion highlighted their role in supporting recent scientific knowledge into IYCF policy.

Other than the NNC, the majority of the government non-health and local NGO sector actors were not found to be influential (Additional file 4). However, even though these sectors (research and academic, local NGO and Government non health) were placed in a non-significant place in the technical support network and majority of them being not influential, most of them were found to be actors who are supportive for IYCF activities in the country (Additional file 3).

Other than actors from above main sectors (government health, development partners, government non health, research and academic, local NGO) involved in IYCF programme and policy process in Sri Lanka, private sector and media were among the 20 actors who received technical inputs from the highest number of other actors (Fig. 1a, In –degree in Additional file 2), which is an advantageous situation for them to function in line within the evidence based recommendations. However, the milk food industry (MFI), which is the industry involved with production of infant formula, and the media were the only actors who had ‘competing interests’ in relation to IYCF activities in Sri Lanka (Fig. 1a). This is a cause for concern as both media and milk food industry were found to be influential actors as well (Additional file 4).

### Communities in the technical support network

A community indicates that nodes belonging to that community have more connections or collaborations among themselves (in terms of technical support or funding) compared to their connections with the nodes belong to other communities. Using the community detection algorithm of ORA software, six communities were identified in the technical support network for IYCF policy decision making in Sri Lanka. Actors of the different communities are indicated by different node shapes (rectangle, circle, triangle etc.) and different colours (Fig. 1b).

All communities were diverse, with actors representing various sectors, such as government health, government non-health, academics etc. Participants highlighted that this indicates smooth flow of technical support and collaboration between actors of various sectors for planning and implementation of IYCF policies and programmes in Sri Lanka, with the most influential actors within each community also being networked across communities. The two largest communities were centred on the FHB and the Ministry of Indigenous Medicine (MIM) respectively. The community analysis also highlighted the significant influence of the Code Monitoring Committee.

### Stakeholders in the funding support network, their level of influence and support

Interviewees identified 36 separate actors at different levels, who involve independently to prioritise how to distribute funds to the recipient institutions, while adhering to the governmental regulations. Therefore Ministry of Finance, development partners and other government and non-government sector institutions are included in the same funding cascade, (Fig. 2a; Additional file 2). Similar to the technical support network, most of the actors in the funding support network as well were from the government health sector.

When analysing the funding support linkages of the government health sector, it was found that they received funds from a large number of sources in different sectors, which is a clue towards the funding adequacy of these institution for IYCF activities (Fig. 2a). Most of the actors from the government health sector were among the top 20 actors for reachability (Fig. 2a, closeness in Additional file 2). Most of the actors from government health sector were seen as acting as gatekeepers for the funding process (Fig. 2a, betweenness in Additional file 2). Specifically, the most influential actors in the government health sector in relation to funding were the Family Health Bureau (FHB) and Ministry of Health (MoH) (Fig. 2a, Additional file 4). The FHB ranked in the top 10 actors for all measures of network centrality, indicating that it both received and provided funding (in degree, out degree), whereas the MoH was highly ranked only for provision of funding. Both were strategically positioned in the network (Fig.2a; betweenness and closeness in additional file 2) and were supportive (Additional file 3).

Since the majority of the government health sector actors were ﻿highly supportive, and the rest were supportive for IYCF programme and policy process in Sri Lanka (Additional file 3) government health sector being gatekeepers in the funding process creates a favourable situation. Most of the actors from the government health sector were influential (Additional file 4). The high level of supportiveness, influence and strategic position of these actors ensures that the fund distribution and utilization happens in a favourable way towards the IYCF policy and programme process in Sri Lanka.

When analysing the funding support linkages of development partners, it was found that almost all of the development partners were providers of funds to other actors (Fig. 2a; Out-degree in Additional file 2) reflecting their active role as donor organizations in Sri Lanka. In particular, WHO, UNICEF, the World Bank and World Food Programme were highly influential through provision of funding (Fig. 2a, Additional file 4). Most of these development partners were also among the top 10 actors with the highest reachability (Fig. 2a; closeness in Additional file 2), which improves the efficacy of the funding process. Further, the development partners were the second most important gatekeepers for the funding process in Sri Lanka (Fig. 2a; betweenness in Additional file 2). All the development partners were also supportive for IYCF programme and policy process in Sri Lanka (Additional file 3). Thus, like the government health sector, development partners also being gatekeepers for the funding process create a favourable situation.

When the government non health and local NGO sectors were considered, the leading role of the Ministry of Finance, which is the topmost institution in the funding process in Sri Lanka, was well reflected in the funding support network (Fig. 2a; Out-degree in Additional file 2). Most of the actors from local NGO sector and government non-health sector received funds from only a limited number of sources (Fig. 2a, In-degree in Additional file 2). They were not reachable (Fig. 2a, closeness in Additional file 2) to other actors in the network and not influential (Additional file 4), creating a less favourable situation for them to receive funds for IYCF related activities, even though most of these actors were ‘supportive’ for IYCF activities in the country (Additional file 3). Similarly, the funding support Net-Map analysis of research and development sector revealed that they received funds from a large number of actors (Fig. 2a, In-degree in Additional file 2), but did not show much degree of capacity to control the flow of funding activities between actors (Fig.2a; Betweenness in Additional file 2).

Other than above main sectors in the funding process in Sri Lanka, private sector was found to be one of the 20 actors who received funds from highest number of sources, and one of the 20 actors with highest reachability. Many of these companies do not oppose the goals of IYCF policy and programme objectives (such as companies involved with supplying goods and printing). Out of all the stakeholders, only the Media and Milk Food Industry were found to have ‘competing interests’.

### Communities in the funding support network

Five communities were identified within the funding support network (Fig. 2b) in Sri Lanka, and actors in different communities are indicated by different shapes and different colours. Most communities had actors representing various sectors such as government health, government non-health, academics etc. (Fig. 2b). This creates a supportive environment for obtaining and utilizing funds for IYCF activities.

### Conclusions and recommendations

This study presents a detailed overview of the IYCF policy landscape in Sri Lanka. The strength of this analysis is in its systematic assessment of IYCF support across a wide spectrum of relevant policy documents, with a focus on best-practice interventions, coupled with a detailed analysis of stakeholder influence on policy and programmes made through one-on-one interviews.

This study found strong, evidence based policy support addressing key IYCF interventions in Sri Lanka. Specific strengths in the policy environment include recognition of the central bodies responsible for IYCF, partnership and networking with other relevant sectors, support for IYCF throughout the health care system, consistent messages on information provision, adequate training for health care workers, provisions for supportive working environment including parental leave, and a focus on equity. However, the lack of mention of IYCF in high-level policy documents suggests that there is an opportunity to further demonstrate political commitment and enhance implementation across sectors by the inclusion of IYCF as a priority in these documents. In addition, there is scope to strengthen provisions related to food security, complementary feeding and the education sector. A review on maternity leave recommendations for employees in different sectors, including the informal sector, would be essential. Having a separate, written policy on IYCF would further strengthen the provision of services by empowering the staff at every level and facilitate supervision and monitoring of IYCF activities in the country.

In the stakeholder analysis, we found that the policies are supported by an influential Government Health sector. The Government Health sector, especially the Family Health Bureau, which is the Maternal and Child Health wing of Ministry of Health, was found to be the technical core. Active involvement of development partners and research and academic sectors improved the policy and programme process through incorporation of international expertise and scientific evidence respectively.

The Ministry of Finance and Ministry of Health, followed by development partners, were found to be the main donors for institutions involved with IYCF activities. Government health and the research and development sectors receive funds from several sources of different sectors and funding bodies were easily accessible for them. One opportunity for strengthening policy arose from the high level of influence of Media and Milk food industry, which have competing interests in pushing forward the IYCF policies. Though adequate provisions are available in policy documents, steps should be taken to address this influence towards a more positive outcome.

Though the research and development sectors provide technical support, they are perceived as less influential. Government non-health and local NGO sectors were technically less connected and less influential. Further, government non-health sector was placed in a less favourable position related to receipt of funds related to IYCF activities. As majority of the actors from these sectors were supportive for IYCF activities in the country, improving their position in the network and level of influence would be an opportunity to enhance the multi-sectoral commitments stressed in policy documents.

Potential limitations of the study are: 1) the Net-Map stakeholder interviews were conducted at workplaces of the informants, which could have influenced their willingness to comment on their own agency; 2) the content analysis was conducted at a single point in time, and thus had a limited capacity to identify changing political will and policy support over time, and 3) only the national level policy documents were reviewed.

In summary, this analysis has identified strong policy support for IYCF in Sri Lanka, facilitated by a diverse and well-connected network of influential stakeholders, led by the government health sector. The findings indicate several opportunities to further strengthen policy support for IYCF through advocacy and strategic stakeholder engagement in Sri Lanka.

## Additional files

Additional file 1: Policy documents relevant to IYCF in Sri Lanka. (DOCX 21 kb)

Additional file 2: Top 10 actors for each measure of IYCF network centrality in Sri Lanka. (DOCX 14 kb)

Additional file 3: Level of support of the actors. (DOCX 13 kb)

Additional file 4: Level of influence of the actors with the highest level of influence. (DOCX 22 kb)

## Abbreviations

AS PHS: Additional Secretary Public Health Services
BCC: Behaviour Change Communication
BMS: Breast Milk Substitute
CCPSL: College of Community Physicians Sri Lanka
CMC: Code Monitoring Committee
DCS: Department of Census and Statistics
DDG PHS 1: Deputy Director (Public Health Services)1
DDG PHS 2: Deputy Director (Public Health Services)2
DEUH: Director/ Estate & Urban Health
DGHS: Director General of Health Services
DHS: Demographic and Health Survey
DI: Director/ Information
DPAD: Director/ Policy Analysis & Development
FCAU: Food Control Administration Unit
FHB: Family Health Bureau
HEB: Health Educational Bureau
Hosp: Hospitals
IA: Independent Academics
IHP: Institute of Health Policy
IYCF: Infant and Young Child Feeding
LMIC: Low and Middle Income Countries
MA: Ministry Agriculture
MAM: Moderate Acute Malnutrition
MCDWA: Ministry of Child Development and Women’s’ Affairs
MCH/FP: Maternal and Child Health /Family Planning
MCN: Maternal and Child Nutrition
MCN/SC: Maternal and Child Nutrition/Sub Committee
MFI: Milk Food Industry
Mfinance: Ministry of Finance
Mfisheries: Ministry of Fisheries
MH: Ministry of Health
MIM: Ministry of Indigenous Medicine
MIT: Ministry Internal Trade
MJ: Ministry Justice
ML: Ministry Labour
MLRDC: Ministry Livestock and Rural community Development
MM: Ministry Media
MMMI: Ministry of Mass Media and Information
MMN: Multiple Micro Nutrients
MOOH: Medical Officers of Health
MRI: Medical Research Institute
NA: Nutrition Alliance
NCD: Nutrition Coordination Division
ND: Nutrition Division
NGOs: Non- Governmental Organizations
NNC: National Nutrition Council
NNP: National Nutritional Policy
NS: Nutrition Society
NSACP: National STD/AIDs Control Programme
NSC: Nutrition Steering Committee
PDMH: Planning Division /Ministry OF Health
PHA: Provincial Health Authorities
PHM: Public Health Midwife
PLWHA: People Living with HIV and AIDs
PS: Private Sectors
PSL: Plan Sri Lanka
PSS: Perinatal Society Sri Lanka
RUTF: Ready to Use Therapeutic Food
SA: Samurdhi Authority
SAH: School and Adolescent Health
SAIFRN: South Asia Infant Feeding Research Network
SAM: Severe Acute Malnutrition
SEARO: South East Asia Regional Office (World Health Organization)
SH: Secretary Health
SLCMA: Sri Lanka College of Medical Administrators
SLCP: Sri Lanka College of Physicians
SLMA: Sri Lanka Medical Association
Sri Lanka Code: Sri Lanka Code for Protection Promotion and Support of Breast Feeding and Marketing of Designated Products
UNICEF: United Nations Children’s Fund
WB: World Bank
WFP: World Food Programme
WHO: World Health Organization
WV: World Vision
