# Supplementary material for: Policy and stakeholder analysis of infant and young child feeding programmes in Sri Lanka
Source: BMC Public Health. 2017 Jun 13;17(Suppl 2):522. doi: 10.1186/s12889-017-4342-4 (PMC5496021; doi:10.1186/s12889-017-4342-4)
Supplement: Supplementary file 1 — Policy documents relevant to IYCF in Sri Lanka. (DOCX 21 kb) [file 12889_2017_4342_MOESM1_ESM.docx]

**Additional file 1: Policy documents relevant to IYCF in Sri Lanka**

| Policy document | Source and Year | General policy Support for IYCF | Provision of evidence based information to mother/caregiver/public | Training of healthcare workers to enhance their capacity | Enable mothers/caregivers to engage with best practice interventions | Other | Details on IYCF in the document |
| --- | --- | --- | --- | --- | --- | --- | --- |
| National  Nutrition policy | MoH  2008 | Yes | Yes | Yes | Yes | Yes | This policy contains provisions on,  -Ensuring political commitment and supportive environment.  -Ensuring food security and safety  -Promoting behaviour towards correct food choices and community empowerment  - Ensuring optimum nutrition through the life cycle, including infants and young children  -Ensuring optimum nutrition of vulnerable populations, after illness with special consideration on PLWHA and during emergencies  - A coordination mechanism involving all stakeholders.  - Establishment of a National Nutrition Surveillance System and generation of evidence |
| Multisector Action Plan on Nutrition | National Nutrition Council  2013 | Yes | Yes | Yes | Yes | Yes | This policy document contains provisions on:  -Installing the Nutrition Coordination Division  -Capacity building of health staff on MCN  -Supplementation programmes for mothers and children  -Review of existing maternity benefits.  - A national advocacy and communication campaign targeting first 1000 days  - Regulations on labelling food items  -Promoting the development new food items with local raw material  - Scaling up of the MMN programme and Zn for the management of diarrhoea. |
| National Strategic Plan on Maternal and Newborn Health | MoH  2011 | Yes | Yes | Yes | Yes | NA | This policy document has provisions on  - Policy level leadership and guidance on MCH  -Upgrading the Family Health Bureau to a centre of excellence in maternal and child health/family planning  - Planning, implementation and monitoring of Maternal and Child --- Health/Family Planning (MCH/FP) programme and ensuring policy support, political support, financial stability and human resources for it.  -Strengthening collaboration among all stakeholders  - Strengthening accessibility and availability of quality maternal and child health services while ensuring equity.  -Improving the management information system on maternal care.  -Ensuring safe motherhood and optimum nutrition status for pregnant mothers |
| National Maternal and Child health Policy | MoH  ,FHB 2012 | Yes | Yes | yes | Yes | Yes | This policy document has identified:  - Safe Motherhood Programme and Promotion of Breastfeeding as some of the key interventions responsible for the reduction in infant and neonatal mortality  - Social and cultural practices as possible causes for difficulty in controlling the child malnutrition  Policy document has provisions on:  -initiating and establishing of exclusive breast feeding for six months and ensuring support by all healthcare professionals  -Protecting, promoting and supporting breastfeeding practices with special emphasis on delivery settings  - Actively promoting evidence based nutrition interventions, especially those targeting mothers and caregivers of children |
| Sri Lanka Code for Protection, Promotion and Support of Breast Feeding and Marketing of Designated Products | MoH  2004 | Yes | Yes | Yes | Yes | Yes | This policy document has provisions on:  -Planning, designing and dissemination of information regarding infant feeding and of the legal control of such information (includes restriction on information provision in marketing and advertising & government commitment to provision of supportive information)  - Prohibition of advertisement or other forms of promotion, or giving of samples as gifts, to both pregnant and breastfeeding women and healthcare workers(includes restriction on activities within facilities) |
| Circular on Change in modality of Management of Children under the age of 5 years with Severe Acute Malnutrition | MoH  2012 | Yes | NA | NA | NA | NA | This policy document recommends  -Managing children with Severe Acute Malnutrition at a hospital with a consultant paediatrician with therapeutic feeding (Ready to Use Therapeutic Food- RUTF)  - Managing the Moderate Acute Malnutrition (MAM) category has to be managed in the field clinics by MOH and field staff with Thriposha as the supplement |
| Infant and Young Child Feeding Guidelines | MoH  2007 | NA | Yes | Yes | Yes | Yes | This policy document has recommendations on:  -timing and duration of BF and CF  - on demand feeding, and feeding with expressed breast milk when necessary  -Age appropriate CF techniques  --Micronutrient and Thriposha supplementation for pregnant and lactating mothers  - Use of fortified complementary food  - Iron , and vitamin A supplementation for infants and young children |
| Maternity Leave –Chapter XII of the Establishment Code | Min. of Public Administration and Home Affairs  2006 | NA | NA | NA | Yes | NA | This policy document has provisions :  -To ensure 84 working days full pay leave in respect of every live child birth and they will not be allowed to resume duty before expiry of 4 weeks after the birth of the child  (All female officers whether permanent, temporary, casual or trainee are entitled to maternity leave under this section) |
| New Schedule for Multiple Micro Nutrient (MMN) Supplementation for 6,12 and 18 month age group | MoH 2012 | NA | NA | NA | NA | Yes | Multiple Micro Nutrient Supplementation should be given in addition to the early introduction of iron rich food |
| The Protocol on Managing Nutritional Problems among Under 5 Children in the Community | MoH  2008 | NA | NA | NA | NA | Yes | This policy document has recommendations on  -Indications for the PHM to refer an infant/ child to the MOH  -Indications to issue Thriposha for infants/children >6 months to 5 years  - Iron supplementation from the age of two months to at least one year (preferably 2 years) for low birth weight and preterm children |
| Guidelines for feeding infants and pre-school children(1-5 years) including orphans and those not living with mothers during an emergency situation | FHB,  MoH  2009 | Yes | Yes | NA | Yes | NA | This policy document has following recommendations  -Promotion of EBF for children less than 6 months and –complementary feeding and Continuation of breast feeding for Infants and young children above 6 months  -Orphaned infants - explore the feasibility to breastfeeding by a surrogate mother, a close relative  - Formula milk is indicated only for orphans or those not living with mother, or weaned off the breast before the emergency  Policy has provisions to   - Protect and support breast feeding in emergency situations - Ensure age appropriate CF and supplements - Prevent promotion of BMS - Replacement feeding when indicated |
| Maternity Benefits ordinance | Min. of labour  1941 | NA | NA | NA | Yes | NA | This policy document has provisions for  - Payment of Maternity Benefits (including maternity leave) to Women Workers and for Others Incidental to the Employment of Such Women before and after their Confinement. |
| MahindaChinthana Vision | 2010 ( no author) | Yes | NA | NA | Yes | ~~NA~~ | This policy document has recommendations to  - Improve the nutrition level of pregnant mothers through promoting good nutrition among pre and during pregnancy and better targeting of food supplementation  -Strengthen the vitamin and mineral supplementation programmes  -Enhance knowledge and promote correct nutritional practices among adolescents and youth  -Improve food security of the poor through raising incomes and food assistance |
| The circular on Sponsorship of Foreign Tours by Manufacturers and Distributors of Designated Products –Circular | MoH  2012 | NA | NA | NA | NA | Yes | This policy document has provisions that:  - Sponsorship of foreign tours to be disclosed and approval of the Monitoring Committee and the Ministry need to be obtained |
| Promotion of Milk powder within Health Institutions under the Ministry of Healthcare and Nutrition –Circular | MoH 2008 | NA | Yes | NA | Yes | NA | This policy document has provisions to  - Protect and Support of Breastfeeding within health institutions  -Prohibit the advertisement of milk food/distribution of free samples/display of milk food company logo within the health institution premises.  -Prohibit the sponsorship from milk food companies  -Prohibit the distribution of any printed material to promote milk powder using the health staff or by other means within health institution premises |
| Breastfeeding: Just 10 steps :The Baby Friendly Way –Circular | MoH  2012 | Yes | Yes | Yes | Yes | NA | This policy document has provisions that  -Every hospital providing maternity services and care for new-born infants should follow the 10 steps to successful breastfeeding  - Every Medical Officer of Health area should follow the Ten steps to successful breastfeeding identified for them. |
| Facilitation of practice of Infant and Young Child Feeding Recommendations on Breast Feeding within Health Institutions | MoH  2012 | NA | NA | NA | Yes | NA | This policy document has provisions that  -Women opting to return to work after first 84 days of paid leave, to be offered one hour nursing break until the baby is 6 months old  - Establishment of Breastfeeding Rooms |
| Shop and Office Employees Act | Min. of Labour  1954 | NA | NA | NA | Yes | NA | This policy document entitles the female employees who are working in work places which are covered by this act to  - paid leave for 14 days before the confinement  - paid leave for 28 days after the confinement  - not to be allowed to carry out any work which may be injurious to her or her child during the 3 months prior to and after the confinement.  - not to terminate the employment by reason of her pregnancy or confinement or of any illness consequent on her pregnancy or confinement.  - not be given the notice of dismissal during absence in accordance with the Act |
| Paternal Leave –Chapter XII of the Establishment Code | Min. of public Admin & home affairs  2006 | NA | NA | NA | Yes | NA | This policy document ensures that  -a permanent, temporary, casual or trainee Public Officer is entitled to a period of 03 working days leave in the occasion of the birth of a child to his wife |
| Guidelines on De-Worming Children and Pregnant Women in Community Setting: | MoH  2012 | NA | NA | NA | NA | Yes | This policy recommends that  -Children under five years (starting at the age of 1 ½ years) from high risk areas to be de-wormed twice a year  -Children under five years (starting at the age of 1^1^/_2_ years) from moderate risk areas to be de-wormed once a year. |
| Vitamin A mega dose supplementation –Revised Schedule | MoH  2009 | NA | NA | NA | NA | Yes | This policy document  - recommends that Vitamin A has to be supplemented every 6 months starting from 6 months of age until 5 years;. then, at the grade 4 and 7 at school.  - describes the contraindications to, and side effects of Vit A  - identifies the authority responsible for implantation of Vit A supplementation new schedule |
| Zinc Supplementation in Managing Diarrhoea among Children Under Five Years of Age | MoH  2013 | NA | NA | NA | NA | Yes | This policy document has recommendations on  - Zn supplementation regime in the management of diarrhoea among children under five years of age |
| Implementation of the Sri Lanka Code for Promotion, Protection and Support of Breast Feeding and Marketing of Designated Products | MoH  2012 | Yes | Yes | Yes | Yes | Yes | This policy document identifies  -Any violation of the provisions of the Code as a cognizable offence under the legal system of Sri Lanka |
| National Strategic Plan Adolescent health(2013-2017) | MoH  2013 | NA | NA | NA | NA | Yes | This policy document recommends that  -The School and Adolescent Health (SAH)program should be implemented in the field by the Medical Officers of Health (MOOH) and their public health staff  - It should include School Medical Inspection, iron folate supplementation for school children, teacher training and educational programmes for health staff |

*NA-Given document has no provisions under this domain*
