# Supplementary material for: Policy and stakeholder analysis of infant and young child feeding programmes in Sri Lanka
Source: BMC Public Health. 2017 Jun 13;17(Suppl 2):522. doi: 10.1186/s12889-017-4342-4 (PMC5496021; doi:10.1186/s12889-017-4342-4)
Supplement: Supplementary file 2 — Top 10 actors for each measure of IYCF network centrality in Sri Lanka. (DOCX 14 kb) [file 12889_2017_4342_MOESM2_ESM.docx]

**Additional file 2: Top 10 actors for each measure of IYCF network centrality in Sri Lanka**

| **Funding** | | | | **Technical support** | | | |
| --- | --- | --- | --- | --- | --- | --- | --- |
| *Out-degree* | *In-degree* | *Betweenness* | *Closeness* | *Out-degree* | *In-degree* | *Betweenness* | *Closeness* |
| MH (0.4444) | PS (0.1944) | MH (0.4308) | MH (0.0160) | FHB (0.5965) | FHB (0.6667) | FHB (0.2766) | FHB (0.0167) |
| UNICEF (0.4167) | CCPSL (0.1667) | UNICEF (0.1942) | FHB (0.0160) | MRI (0.4737) | MIM (0.4211) | MH (0.0732) | MRI (0.0116) |
| WHO (0.3333) | SLCMA (0.1667) | FHB (0.1501) | PS (0.0150) | MH (0.4386) | DDG PHS 1 (0.2632) | MRI (0.0398) | MH (0.0115) |
| WB (0.2222) | FHB (0.1389) | PS (0.1220) | UNICEF (0.0140) | HEB (0.2105) | CMC (0.1754) | MIM (0.0322) | MIM (0.0111) |
| WFP (0.2222) | MRI (0.1389) | WHO (0.1197) | WHO (0.0140) | WHO (0.2105) | Media (0.1754) | WHO (0.0302) | WHO (0.0106) |
| FHB (0.1389) | MIM (0.1389) | DI (0.1048) | CCPSL (0.0140) | UNICEF (0.1754) | UNICEF (0.1579) | HEB (0.0104) | HEB (0.0105) |
| MFinance (0.1111) | IA (0.1111) | MFinance (0.0623) | SLCMA (0.0140) | IA (0.1579) | NNC (0.1579) | DDG PHS 1 (0.0087) | DDG PHS 1 (0.0103) |
| SH (0.0833) | MFisheries (0.1111) | DGHS (0.0540) | MRI (0.0130) | SH (0.1579) | PS (0.1579) | SH (0.0084) | UNICEF (0.0103) |
| DDG PHS 2 (0.0556) | Universities (0.1111) | MRI (0.0436) | NSACP (0.0130) | CCPSL (0.1404) | MRI (0.1404) | UNICEF (0.0078) | WFP (0.0099) |
|  | Hosp. (0.0278) | NA (0.0131) | DDG PHS 1 (0.0110) | WV (0.0877) | WB (0.0877) | WB (0.0017) | SEARO (0.0094) |

|  | Government Health |  | Development Partners |  | Research and Academic |  | Government Non Health |  |  | Local NGO |  | Other |
| --- | --- | --- | --- | --- | --- | --- | --- | --- | --- | --- | --- | --- |
|  |  |  |  |  |  |  |  |  |  |  |  |  |
