# Supplementary material for: Policy and stakeholder analysis of infant and young child feeding programmes in Sri Lanka
Source: BMC Public Health. 2017 Jun 13;17(Suppl 2):522. doi: 10.1186/s12889-017-4342-4 (PMC5496021; doi:10.1186/s12889-017-4342-4)
Supplement: Supplementary file 4 — Level of influence of the actors with the highest level of influence. (DOCX 22 kb) [file 12889_2017_4342_MOESM4_ESM.docx]

**Additional file 4: Level of support of the actors**
